# Happy Family Kitchen II: a cluster randomized controlled trial of a community-based positive psychology family intervention for subjective happiness and health-related quality of life in Hong Kong

**DOI:** 10.1186/s13063-016-1508-9

**Published:** 2016-07-29

**Authors:** Henry C. Y. Ho, Moses Mui, Alice Wan, Yin-lam Ng, Sunita M. Stewart, Carol Yew, Tai Hing Lam, Sophia S. Chan

**Affiliations:** 1 School of Public Health, The University of Hong Kong, 5/F William M. W. Mong Block, 21 Sassoon Road, Pokfulam, Hong Kong; 2 Service Development, The Hong Kong Council of Social Service, Wanchai, Hong Kong; 3 Department of Psychiatry, The University of Texas Southwestern Medical Center at Dallas, Dallas, TX USA; 4 United Centre of Emotional Health and Positive Living, United Christian Nethersole Community Health Service, Kowloon, Hong Kong; 5 School of Nursing, The University of Hong Kong, 4/F William M. W. Mong Block, 21 Sassoon Road, Pokfulam, Hong Kong

**Keywords:** Randomized controlled trial, Community-based intervention, Positive psychology, Subjective happiness, Subjective well-being, Health-related quality of life

## Abstract

**Background:**

Most positive psychology interventions conducted in the West have been focused on the individual. Family relationships are highly valued in the Chinese collectivist culture, and it is of interest to know whether family-focused interventions can improve the well-being of Chinese people. We have previously reported the effectiveness of a positive psychology family intervention in terms of family well-being. Based on the data derived from the Happy Family Kitchen II project, this paper examines the effectiveness of a community-based positive psychology family intervention on subjective happiness and health-related quality of life.

**Methods:**

Thirty-one social service units and schools organized intervention programs for 2070 participants in Hong Kong. In a cluster randomized controlled trial, participants were randomly assigned on the basis of computer-generated numbers into the intervention group or the control group. The intervention programs emphasized one of five positive psychology themes: joy, gratitude, flow, savoring, and listening. The control group engaged in activities unrelated to the intervention, such as arts and crafts workshops. Subjective happiness and mental and physical quality of life were assessed at baseline and at 4 weeks and 12 weeks postintervention.

**Results:**

Data of 1261 participants were analyzed. The results showed that the intervention was more effective than the control condition in improving subjective happiness, with a small effect size, at 12 weeks postintervention (β = .15, *p* = .020, Cohen’s *d* = .16). However, there were no improvements in mental and physical quality of life in the intervention group compared with the control group at 4 weeks (β = .39, *p* = .494, *d* = .05; β = −.10, *p* = 1.000, *d* = −.01, respectively) and 12 weeks postintervention (β = .71, *p* = .233, *d* = .08; β = −.05, *p* = 1.000, *d* = −.01, respectively). Furthermore, the booster session was no more effective than the tea gathering session in improving subjective happiness (β = .00, *p* = .990, *d* = .00) or mental (β = 1.20, *p* = 1.000, *d* = −.04) and physical quality of life (β = .15, *p* = 1.000, *d* = −.01).

**Conclusions:**

The analyses extend previous findings of salutary effects on family well-being by showing that positive psychology family interventions can improve subjective happiness. Suggestions for future research are proposed.

**Trial registration:**

ClinicalTrials.gov NCT01796275. Retrospectively registered 19 February 2013.

**Electronic supplementary material:**

The online version of this article (doi:10.1186/s13063-016-1508-9) contains supplementary material, which is available to authorized users.

## Background

Families residing in urban areas often face incompatible demands between work and family responsibilities, which can interfere with family communication and gatherings that are essential to the well-being of family members [1–3]. Therefore, a series of intervention studies under the umbrella of the FAMILY Project, including Happy Family Kitchen (HFK I) and Happy Family Kitchen II (HFK II), have been conducted to develop, implement, and evaluate a community-based positive psychology family intervention for improving family communication and family well-being in Hong Kong. These interventions were developed for families on the basis of positive psychology concepts applied to a cooking and dining setting. Based on use of a longitudinal methodology, results from HFK I provided initial support for the intervention program in improving family communication and family well-being [4]. Data derived from a cluster randomized controlled trial (cRCT) in HFK II provided further support with greater improvements in family health and family happiness in the intervention group than in the control group [5]. Extending our previous work, in the present paper we report findings on the individual-level outcomes of HFK II to examine the effectiveness of a positive psychology family intervention on subjective happiness and mental and physical quality of life.

Research done in the West has consistently provided evidence for the effectiveness of positive psychology interventions in terms of subjective well-being, such as life satisfaction, resilience, hope, and positive affect, and in reducing negative affect and pessimism [6–9]. Furthermore, the positive effect of positive psychology interventions extends to mental health: depressive symptoms have been alleviated in patients with depression as well as in individuals in the general population [9–12]. These interventions have typically involved some form of individual-based exercises to improve individual-level outcomes. The exercises can include imagining the best overall possible future (optimism), recalling positive events for which one is thankful (gratitude), and writing about the happy moments of life (joy) [13]. Family relationships are highly valued in the Chinese collectivist culture. In particular, family goals such as maintaining harmonious family relationships are valued over individual desires [14]. Family contact, quality, and functioning are important for the subjective well-being of the Chinese [15–17]. As we have previously reported the salutary effects of positive psychology family interventions on family well-being [5], a major contribution of the present paper is to evaluate the effect on individual-level outcomes, including subjective happiness as a measure of subjective well-being and the mental and physical components of health-related quality of life. To our knowledge, no similar studies have used a large-scale cRCT to examine the effectiveness of a community-based positive psychology family intervention on subjective happiness and mental and physical quality of life.

Although there is an abundance of positive psychology research from the West on subjective well-being and, to a lesser extent, mental health, very few of these studies have specifically examined physical health as an intervention outcome. A positive emotion regulation intervention was able to enhance not only subjective well-being and mental health but also physical health, such as reduced headache, stomachache, sleep problems, and cramps [18]. Another study showed that writing about positive experiences reduced the number of clinic visits for illness [19]. A comprehensive review of the literature found that the effectiveness of interventions designed to enhance physical health among ill populations through positive cognitions and attitudes, such as optimism and gratitude, was encouraging though not definitive [13]. Only one relevant study with a Chinese sample showed that a positive psychology stress management training program was effective for improving both psychological and physical symptoms of organizational stress in Hong Kong [20]. Although there has been minimal study of positive psychology interventions for promoting physical health, related psychosocial interventions have provided evidence for improving physical functioning and the immune system and for alleviating the symptoms of chronic illnesses [21–23]. Furthermore, a meta-analysis of findings derived from 150 experimental, ambulatory, and longitudinal studies demonstrated that subjective well-being has a positive effect on physical health, including short-term outcomes (e.g., immune system response and pain tolerance), long-term outcomes (e.g., cardiovascular functioning and respiratory functioning), and disease and symptom control (e.g., respiratory control and disease progression) [24]. Therefore, physical health might be one of the life domains that can be influenced by interventions aimed at promoting subjective well-being. Taken together, the evidence suggests that our intervention might be effective for enhancing the physical quality of life, even though it is psychosocially oriented.

In the design of the intervention, feasibility, practicality, acceptability, and sustainability were taken into consideration. Because the intervention was applied to a community setting, cooking and dining were chosen to provide a platform for family-based activities so that the intervention was feasible even for people with busy working lives. This was the most practical in Hong Kong because it is common for Chinese people to cook and dine with family members [25, 26]. To enhance the intervention’s acceptability to busy families, it was brief, with only one core session and one booster session, thus minimizing the burden placed on the service recipients and maximizing the retention rate. For sustainability, the brief nature of the intervention minimized program implementation costs, and the use of community venues, including social service units and schools, ensured that a large scope of community beneficiaries could be covered. To date, these intervention attributes are uncommon in the literature.

To foster positive communication among families in Hong Kong, five positive psychology themes, namely joy, gratitude, flow, savoring, and listening, were chosen for the intervention framework [5]. The “joy” theme was focused on discovering the short-term pleasures and long-term gratifications of positive family interactions [27]. The intervention involved sharing and reminiscing about happy experiences with family members and creating more enjoyable moments by cooking and dining together. The “gratitude” theme emphasized the importance of thanking and appreciating family members for their contribution and support [28]. The participants were encouraged to appreciate and express gratitude to their family members for contributing to family meals and other household chores. The aim of the “flow” theme was to engage family members in engrossing and enjoyable activities that could facilitate interaction, cooperation, and the identification of character strengths [29, 30]. Specifically, the participants were encouraged to prepare a family meal together, to stay focused during the activity, and to observe and identify each other’s strengths, such as curiosity, generosity, and teamwork. The “savoring” theme emphasized the importance of cherishing the enjoyable moments of family gatherings [11]. In particular, slowing down the pace of eating, savoring the food prepared by family members, and treasuring the good times during family meals were encouraged. The “listening” theme involved understanding and addressing the emotional needs of family members [30]. Participants were encouraged to actively listen to their family members when they spoke, to observe their nonverbal expressions, and to show interest in and acceptance of their emotions and concerns.

cRCT was adopted in the present study to examine the effects of a positive psychology family intervention on subjective happiness and mental and physical quality of life. The rationale for using a cluster design included the community-based nature of the intervention, the reduced risk of experimental contamination, and the efficacy of participant recruitment. The Consolidated Standards of Reporting Trials (CONSORT) cluster trials checklist shows this in more detail (Additional file 1). It was hypothesized that, at the individual participant level, participants in the intervention arm would show improvements in subjective happiness and mental and physical quality of life compared with the tea gathering control group (hypothesis 1 [H1]), as measured over time at baseline and at 4 weeks and 12 weeks postintervention, and that, after the core session, the booster intervention arm would show more improvements than the no-booster intervention arm in subjective happiness and mental and physical quality of life at 12 weeks postintervention (hypothesis 2 [H2]).

## Methods

### Participants

The HFK II project [5] was implemented in two districts in Hong Kong, namely Tsuen Wan and Kwai Tsing, from May 2012 to June 2013. The inclusion criteria at the individual participant level were as follows: (1) residents, service users, or students in the Tsuen Wan or Kwai Tsing district; (2) attend the program sessions with one or more family members; (3) at least one family member is aged 18 years or older and the accompanying family member(s) is aged 6 years or older; and (4) able to communicate in Chinese. The inclusion criteria at the cluster level were as follows: (1) social service organizations and schools in the Tsuen Wan or Kwai Tsing district, (2) social service workers and teachers from each participating social service organization and school attend a train-the-trainer workshop and implement subsequent community programs, and (3) participating social service workers and teachers must be able to communicate in Chinese. Participants were recruited from local social service units, primary and secondary schools, and the government’s social welfare department. A sample size of at least 1920 participants was required for this study to detect small effect sizes of .20 with a statistical power of .80, an α of .05, and an attrition rate of 50 % [31]. In total, 2513 individuals were invited, and 2070 eligible participants from 973 families participated in the study.

All potential participants were recruited by the participating service organizations and schools through various means, including (1) phone invitations; (2) promotional materials such as posters, banners, leaflets, and publications; (3) promotion through e-mails and websites; (4) face-to-face invitations; (5) social service workers’ and teachers’ referrals; (6) outreach recruitment on the streets; and (7) home visits. Written consent was obtained from each participant prior to participation in the programs. For children enrolled in the study, written consent was obtained from their next of kin, caretakers, or guardians on their behalf. Participation was completely voluntary, and participants had the right to withdraw at any time without consequences. As an incentive for completing all four questionnaires, two HK$50 (approximately US$13) supermarket gift vouchers were given to each family at the end of the study. Ethics approval was granted by the institutional review board of the University of Hong Kong/Hospital Authority Hong Kong West Cluster (UW 12-502). This study is registered with ClinicalTrials.gov (NCT01796275).

**Fig. 1:**
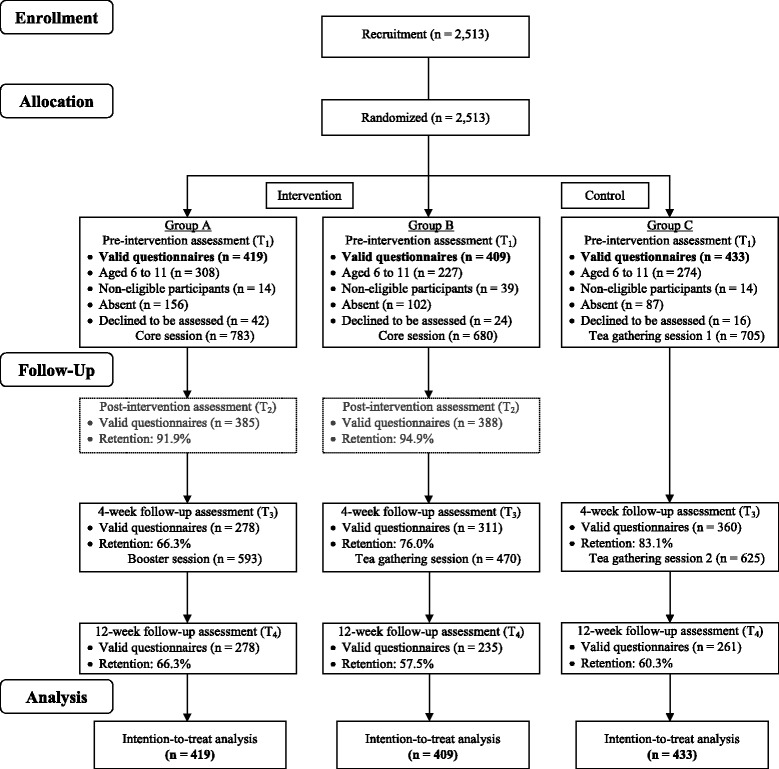
Consolidated Standards of Reporting Trials (CONSORT) flow diagram of participants in Happy Family Kitchen II trial through each stage of the study [5]. The core program contents of groups A and B were identical. Subjective happiness and mental and physical quality of life were not assessed immediately postintervention

The study CONSORT flow diagram is presented in Fig. 1. As shown in the flow diagram, participants aged 6–11 years (n = 809) were excluded from analysis because many of them reported difficulty in understanding the questionnaire items. Of the 1261 participants who met the criteria, 954 (75.7 %) were women, 528 (43.2 %) were aged between 35 and 44 years, and 870 (71.5 %) reported attaining a secondary education level.

### Procedures

Social service workers and teachers were given sufficient knowledge and skills in a train-the-trainer workshop, which was delivered by professional academics and psychologists, to design and implement the community programs. The workshop, conducted between May and September 2012, covered the five themes of positive psychology, the program design, and the program evaluation. A training kit was distributed to each trainee as a practical guide for planning the community programs.

As defined by the HFK II protocol, the cRCT was used to randomly allocate 31 participating service units and schools (the clusters) in the Tsuen Wan and Kwai Tsing districts into three groups by using computer-generated random numbers (allocation ratio of 1:1:1). An independent statistician who had no contact with the service units, schools, or participants performed the randomization and allocation. The participants were not informed about the other interventions that were available in this study. For group A (intervention arm 1, consisting of 11 clusters), the service units and schools delivered a core session of at least 2 h, followed by a booster session of at least 1 h 4 weeks later. For group B (intervention arm 2, consisting of 10 clusters), the service units and schools also delivered a core session of at least 2 h, followed by a tea gathering session 4 weeks later. For group C (wait list control consisting of ten clusters), the service units and schools delivered a tea gathering session at the beginning and 4 weeks later. The core session involved group activities in one of five positive psychology themes, as described in the Background section above; the booster session was focused on consolidating the knowledge and skills obtained from the core session; and the tea gathering session covered topics unrelated to the intervention, such as arts and crafts workshops [5]. The group activities were delivered to an average of 31 families together per group, with the activities focused on the family as a unit. The context of the group activities was determined by the social service workers and teachers to fit the needs of their programs and specific service recipients. The majority of the group activities were held indoors, such as in social service centers and schools (88.4 %), and the others were held outdoors, such as at campsites and parks (11.6 %).

Questionnaires on individual participant-level outcomes were administered at preintervention (baseline assessment), immediate postintervention (immediately after the core session for groups A and B only), 4 weeks after the baseline assessment (before the booster session in group A, before the tea gathering in group B, and before the second tea gathering in group C), and 12 weeks after the baseline assessment. However, immediate postintervention assessment was used only to assess the primary outcomes of family communication and family well-being as well as the participants’ evaluations of the core session. Subjective happiness and health-related quality of life reported in this paper were assessed at baseline and at 4 weeks and 12 weeks postintervention to minimize the burden of multiple lengthy questionnaires during the core session. Furthermore, the intervention program was intended to promote longer-term effects at 4 weeks and 12 weeks postintervention rather than immediately after the core session. The retention rate at each time point was as follows: that of group A was 91.9 % immediately postintervention, 66.3 % at 4 weeks postintervention, and 66.3 % at 12 weeks postintervention; that of group B was 94.9 % immediately postintervention, 76.0 % at 4 weeks postintervention, and 57.5 % at 12 weeks postintervention; and that of group C was 83.1 % at 4 weeks postintervention and 60.3 % at 12 weeks postintervention (Fig. 1). The reasons for nonparticipation, as reported by the participants, included illness, other personal commitments, being out of town, and unfavorable weather.

### Intervention program

This study equitably involved academic researchers, social service workers, and teachers throughout the research process. Although such collaboration requires mutual effort and time, it can result in substantial benefits to all parties. The collaborative involvement of academic and community institutions facilitated knowledge exchange between professionals, the recruitment of participants, the delivery of the large-scale intervention programs, the collection of data, and the dissemination of evidence-based approaches [32]. Furthermore, the tools of community engagement and involvement enabled the service units and schools to develop the programs according to the characteristics and needs of their participants. A total of 31 community programs were implemented within the period from August 2012 to June 2013. Social service workers and teachers who received the train-the-trainer workshop planned and implemented the community programs with a focus on one of the five positive psychology themes of their choice. The themes were chosen by the social service workers and teachers on the basis of their experience with the needs of their particular service recipients. The choices of themes in groups A and B were as follows: six on joy, three on gratitude, four on flow, four on savoring, and four on listening. A standardized protocol, approved by a project steering committee consisting of academic researchers and managerial staff from the participating agencies, guided the aim of the intervention: to provide a platform for positive communication by cooking and dining together with family members to enhance the participants’ well-being. In line with the positive psychology approach, this intervention was preventive in nature by promoting positive quality and capacity in individuals and families [33–35], including the enhancement of subjective happiness and health-related quality of life.

To ensure adherence to the guiding principles so that the consistency and quality of the community programs were maximized, the participating service units and schools submitted program proposals to the project steering committee, received comments and made improvements accordingly, and then were awarded funding to implement the proposals. A research assistant was present in each session (core session, booster session, and tea gathering) to monitor and evaluate the process of the intervention. The observation involved assessing the fidelity (measured by the adherence to program guidelines: “Overall speaking, the degree of adherence to the proposal is ___%”), the dose delivered (measured by the duration of the program delivery: “This program spent around ___ minutes on core message delivery”), and the dose received (measured by the participants’ interest, involvement, and satisfaction in the program: “The extent of participants’ interest/involvement/satisfaction in the program: 1 = *very low*, 5 = *very high*”). Overall, 81.8 % and 72.7 % of the community programs were implemented in accordance with the specified fidelity and dosage, respectively. On a 5-point scale, the participants’ interest (mean = 4.10, SD = .59), involvement (mean = 4.16, SD = .68), and satisfaction (mean = 4.14, SD = .58) in the programs were high [5].

### Outcome measures

#### Subjective happiness

Subjective happiness was assessed using the Subjective Happiness Scale [36], which consists of four items that require responses on a 7-point scale (e.g., 1 = *less happy*, 7 = *more happy*), with a higher total score indicating a higher level of happiness. An example item is “Compared to most of my peers, I consider myself more happy.” The Chinese version of the scale has been validated in Hong Kong [37]. In the present study, the scale has a high degree of reliability (intraclass correlation coefficient [ICC] .79; 95 % CI .761, .813).

#### Health-related quality of life

Health-related quality of life was assessed using the Short Form Health Survey (SF-12v2) [38]. This instrument consists of 12 items on mental and physical quality of life. Depending on the question, responses are given on a 3-point scale (e.g., 1 = *yes, limited a lot*; 3 = *no, not limited at all*) or on a 5-point scale (e.g., 1 = *not at all*, 5 = *extremely*). An example question is, “During the past 4 weeks, how much did pain interfere with your normal work (including both work outside the home and housework)?” Mental component summary and physical component summary scores were calculated using the standard scoring algorithms, which involved weighting sums of the domain scale scores with the factor scoring coefficients as described in the SF-12v2 manual [39]. The Chinese version of the scale has been validated in local populations [38, 40]. In the present study, the scale has a high degree of reliability for the mental component (ICC.81; 95 % CI .781, .832) and physical component (ICC .82; 95 % CI .797, .844).

### Data analysis

To examine whether the cluster randomization resulted in comparability among the groups, analysis of variance was conducted with the intervention and control groups as the independent variable and the outcome measures at baseline as dependent variables. Pearson’s χ^2^ tests were conducted to compare the demographic characteristics between the groups. To examine the effectiveness of the community programs, random effects linear models were constructed using WinBUGS [41]. The models were fitted to the outcomes of interest (i.e., subjective happiness and health-related quality of life) for the three groups (i.e., groups A, B, and C) and three time points (i.e., baseline and 4 weeks and 12 weeks postintervention) while the confounding effects of age, sex, and education level were controlled. In addition, individual correlations across time, correlations among family members, and the cluster effect of individuals in the same program were analyzed as random effects with different variances. These analytical procedures were adopted to assess whether there were differences in the outcome changes between the intervention and control groups (i.e., A and B vs. C) and whether the booster session had any effect on the outcome measures compared with the tea gathering session after the core session (i.e., A vs. B at 12 weeks postintervention). For intention-to-treat analysis [42], missing observations were treated with full maximum likelihood inference, which assumes that the data were missing at random (i.e., individuals with missing data were assumed to respond similarly to individuals with complete data and similar demographics) [43]. This assumption is preferred over baseline or last observation carried forward analysis because of reduction in biases, which produces more reliable estimates [44]. Little’s missing completely at random (MCAR) test revealed that the data were MCAR (χ^2^ = 139.02, *df* = 129, *p* = .258), suggesting that there were no identifiable patterns within the missing data. Effect sizes were estimated on the basis of posterior distribution obtained from Bayesian analysis [45]. A major advantage of the Bayesian approach is the ability to fit complex statistical models to a broad variety of datasets and to produce meaningful and comprehensive information about the estimates, including the means, SDs, effect sizes, and differences between groups [45, 46]. An effect size (Cohen’s *d*) of .2 was considered as a small effect, .5 as a medium effect, and .8 or above as a large effect [47]. This effect size measure has also been adopted in other randomized controlled trials in which repeated measures were analyzed using mixed linear models with full maximum likelihood inference [48]. Sensitivity analysis with the inclusion of participants aged 6–11 years produced similar results for all hypotheses (data not shown).

## Results

As shown in Table 1, no statistically significant differences were detected among groups A, B, and C on the baseline scores of subjective happiness [*F*(2, 1149) = .24, *p* = .789] and mental [*F*(2, 1045) = .04, *p* = .963] and physical quality of life [*F*(2, 1045) = .91, *p* = .405]. Therefore, cluster randomization resulted in comparability in the outcome measures among the intervention and control groups. For the demographic characteristics, age [χ^2^(10) = 56.43, *p* < .001], sex [χ^2^(2) = 7.03, *p* = .030], and education level [χ^2^(6) = 25.53, *p* < .001] were significantly different among the three groups, thus justifying the need to statistically control for these variables in the main analyses.

**Table 1:** Demographic characteristics and outcome measures at baseline by group

	Group A (n = 419)	Group B (n = 409)	Group C (n = 433)	*p* Value
Age,^a,b^ years				.000
12–17	30 (7.3)	50 (12.7)	27 (6.5)	
18–34	60 (14.6)	95 (24.1)	118 (28.4)	
35–44	195 (47.3)	147 (37.3)	186 (44.8)	
45–54	77 (18.7)	72 (18.3)	52 (12.5)	
55–64	24 (5.8)	24 (6.1)	23 (5.5)	
65 or older	26 (6.3)	6 (1.5)	9 (2.2)	
Sex^a,b^				.030
Male	90 (21.5)	118 (28.9)	98 (22.7)	
Female	329 (78.5)	291 (71.1)	334 (77.3)	
Education level^a,b^				.000
None	9 (2.2)	7 (1.8)	6 (1.4)	
Primary	76 (18.6)	50 (12.7)	49 (11.8)	
Secondary	276 (67.5)	269 (68.4)	325 (78.5)	
Tertiary or above	48 (11.7)	67 (17.0)	34 (8.2)	
Subjective happiness^c,d^ (4 items, 1–7 scale)	4.67 (1.10)	4.72 (1.13)	4.70 (1.13)	.79
Health-related quality of life^c,d^ (12 items, 0–100 scale)				
Mental component	44.77 (8.75)	44.89 (9.20)	44.70 (8.94)	.96
Physical component	47.01 (8.23)	47.65 (7.06)	47.71 (7.84)	.41

*Note.* Younger participants (aged < 12 years) were excluded

^a^ n (%)

^b^ *p* Values were derived from Pearson’s χ^2^ test

^c^ Mean (SD)

^d^ *p* Values were derived by analysis of variance

**Fig. 2:**
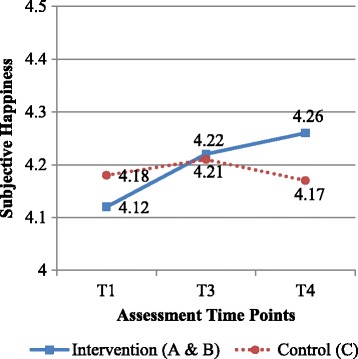
Effectiveness of the intervention group in terms of subjective happiness compared with the control group

With regard to the effectiveness of the intervention (Table 2), the increase in subjective happiness at 12 weeks postintervention was significantly greater in the intervention group than in the control group (β = .15, SD = .06, *p* = .020, *d* = .16, 95 % CI .024, .271) (Fig. 2). The model fit for subjective happiness was σ^2^ = .62. However, changes in mental quality of life in the intervention group did not significantly differ from those in the control group at 4 weeks (β = .39, SD = .57, *p* = .494, *d* = .05, 95 % CI −.719, 1.505) and 12 weeks postintervention (β = .71, SD = .60, *p* = .233, *d* = .08, 95 % CI −.447, 1.884). Changes in physical quality of life in the intervention group also did not significantly differ from those in the control group at 4 weeks (β = −.10, SD = .48, *p* = 1.000, *d* = −.01, 95 % CI −1.029, .835]) and 12 weeks postintervention (β = −.05, SD = .50, *p* = 1.000, *d* = −.01, 95 % CI −1.025, .933]). The model fit for mental and physical quality of life was σ^2^ = 5.66 and 4.74, respectively.

**Table 2:** Changes in groups A and B compared with group C in subjective happiness and health-related quality of life

	Time point	Groups A and B, mean (SD)	Group C, mean (SD)	β (SD)	*d* ^a^	95 % CI	
						LL	UL
Subjective happiness (4 items, 1–7 scale)	Baseline	4.12 (.16)	4.18 (.17)	–	–	–	–
	4 weeks	4.22 (.16)	4.21 (.17)	.07 (.06)	.08	−.044	.187
	12 weeks	4.26 (.16)	4.17 (.17)	.15 (.06)	.16^b^	.024	.271
Health-related quality of life (12 items, 0–100 scale)							
Mental component	Baseline	38.68 (1.26)	39.26 (1.32)	–	–	–	–
	4 weeks	39.51 (1.26)	39.69 (1.32)	.39 (.57)	.05	−.719	1.505
	12 weeks	39.86 (1.27)	39.72 (1.34)	.71 (.60)	.08	−.447	1.884
Physical component	Baseline	50.62 (1.04)	50.94 (1.07)	–	–	–	–
	4 weeks	50.38 (1.04)	50.80 (1.07)	−.10 (.48)	−.01	-1.029	.835
	12 weeks	50.91 (1.05)	51.27 (1.08)	−.05 (.50)	−.01	-1.025	.933

*LL* lower limit, *UL* upper limit

The estimates for the covariates of age, sex, and education were as follows. That of subjective happiness was β = .08; SD = .02; 95 % CI, .028, .123; β = .02; SD = .07; 95 % CI, −.110, .158; β = .05; SD = .02; 95 % CI, .005, .092, respectively. That of mental quality of life was β = .79; SD = .19; 95 % CI, .413, 1.169; β = .91; SD = .54; 95 % CI, −.146, 1.954; β = .45; SD = .17; 95 % CI, .113, .791, respectively. That of physical quality of life was β = −1.095; SD = .17; 95 % CI, −1.420, −.767; β = 1.53; SD = .49; 95 % CI, .570, 2.487; β = .51; SD = .15; 95 % CI, .220, .803, respectively)

*Note.* Younger participants (aged <12 years) were excluded

^a^ Cohen’s *d* (small = .20; medium = .50; large = .80)

^b^ *p* < .05

For within-group changes across time points (Table 3), subjective happiness in the intervention group significantly increased from baseline to 4 weeks (β = .10, SD = .04, *p* = .005, *d* = .20, 95 % CI .030, .167) and 12 weeks postintervention (β = .14, SD = .04, *p* < .001, *d* = .27, 95 % CI .068, .209). The mental quality of life in the intervention group also significantly increased from baseline to 4 weeks (β = .82, SD = .33, *p* = .013, *d* = .17, 95 % CI .174, 1.472) and 12 weeks postintervention (β = 1.17, SD = .34, *p* = .001, *d* = .24, 95 % CI .518, 1.834). However, the physical quality of life in the intervention group did not significantly increase from baseline to 4 weeks (β = −.24, SD = .28, *p* = 1.000, *d* = −.06, 95 % CI −.784, .306) and 12 weeks postintervention (β = .29, SD = .28, *p* = .307, *d* = .07, 95 % CI −.263, .841). There were no significant increases in the control group on all outcome measures at 4 weeks and 12 weeks postintervention.

**Table 3:** Changes in groups A and B on subjective happiness and health-related quality of life

	Time Point	Groups A and B, mean (SD)	β (SD)	*d* ^a^	95 % CI	
					LL	UL
Subjective happiness (4 items, 1–7 scale)	Baseline	4.12 (.16)	–	–	–	–
	4 weeks	4.22 (.16)	.10 (.04)	.20^b^	.030	.167
	12 weeks	4.26 (.16)	.14 (.04)	.27^c^	.068	.209
Health-related quality of life (12 items, 0–100 scale)						
Mental component	Baseline	38.68 (1.26)	–	–	–	–
	4 weeks	39.51 (1.26)	.82 (.33)	.17^d^	.174	1.472
	12 weeks	39.86 (1.27)	1.17 (.34)	.24^c^	.518	1.834
Physical component	Baseline	50.62 (1.04)	–	–	–	–
	4 weeks	50.38 (1.04)	−.24 (.28)	−.06	−.784	.306
	12 weeks	50.91 (1.05)	.29 (.28)	.07	−.263	.841

*LL* lower limit, *UL* upper limit

The estimates for the covariates of age, sex, and education were as follows. That of subjective happiness was β = .08, SD = .02, 95 % CI .028, .123; β = .02, SD = .07, 95 % CI −.110, .158; β = .05, SD = .02, 95 % CI .005, .092, respectively. That of mental quality of life was β = .79, SD = .19, 95 % CI .413, 1.169; β = .91, SD = .54, 95 % CI −.146, 1.954; β = .45, SD = .17, 95 % CI .113, .791, respectively. That of physical quality of life was β = −1.095, SD = .17, 95 % CI −1.420, −.767; β = 1.53, SD = .49, 95 % CI .570, 2.487; β = .51, SD = .15, 95 % CI .220, .803, respectively

*Note.* Younger participants (aged <12 years) were excluded

^a^ Cohen’s *d* (small = .20; medium = .50; large = .80)

^b^ *p* < .01

^c^ *p* < .001

^d^ *p* < .05

For the effectiveness of the booster session, subjective happiness (β = .00, SD = .07, *p* = .990, *d* = .00, 95 % CI −.144, .147) and mental (β = 1.20, SD = .67, *p* = 1.000, *d* = −.04, 95 % CI −.110, 2.506) and physical quality of life (β = .15, SD = .56, *p* = 1.000, *d* = −.01, 95 % CI −.954, 1.259) were not significantly different between groups A and B at 12 weeks postintervention. The model fit for subjective happiness and mental and physical quality of life was σ^2^ = .64, 5.78, and 4.87, respectively.

## Discussion

The intervention was more effective than the control group in improving subjective happiness, with a small effect size, at 12 weeks postintervention. However, there were no improvements in mental and physical quality of life in the intervention group compared with the control group. Thus, the results only partially supported H1. Furthermore, the booster session was no more effective than the tea gathering session in improving subjective happiness and mental and physical quality of life after the core session, thus supporting the rejection of H2.

The positive finding on subjective happiness concurs with prior research on the positive psychology intervention, which was effective for a number of subjective well-being outcomes [6–9]. With the involvement of family members in the intervention program and the emphasis on family communication and family activities, the present study reveals that the positive psychology intervention can also enhance subjective well-being in a collectivistic community. These findings indicate that positive psychology exercises, which have typically been oriented toward individuals, can be modified and tailored to families. Future research should consider examining other individual-level outcomes of family-based interventions, such as life satisfaction and positive and negative affect.

Our positive psychology family intervention was also found to have a selective influence on individual-level outcomes, with improvements in subjective well-being but not health-related quality of life. This suggests that the currently available techniques in positive psychology may not be effective for improving health status. This contrasts with some of the research findings reported in the literature, which provide evidence for the positive effect of positive psychology interventions on mental health [9–12] and physical health [13, 18, 19]. However, the literature on positive psychology interventions is not consistent regarding their effects on health outcomes. In a replication study of Seligman and colleagues’ work, it was found that positive psychology exercises, which included writing about three good things in life and using signature strengths in a new way, did not produce greater reductions in depression over time compared with the control group, although a positive result for happiness was found [49]. Furthermore, a cross-sectional study on character strengths, which were adapted in the theme-based interventions in the present study, revealed that the use of strengths is positively predictive of subjective well-being, but it did not predict the mental or physical components of health-related quality of life [50]. A possible reason may be that health-related quality of life is more stable over time than subjective well-being is, especially among healthy individuals, and is therefore less responsive to positive psychology interventions and positive behaviors. An alternative explanation may be that the target behaviors of these interventions were more relevant to subjective well-being. Future studies that modify the positive psychology intervention by tailoring to behavioral changes in physical exercise and healthy diet may improve the physical quality of life as well. In particular, positive psychology concepts can be used to promote healthy behaviors such as discovering the joy of physical exercise or savoring a healthy dish. However, it is noteworthy that within-group improvement in mental quality of life was observed in the intervention group at 4 weeks and 12 weeks postintervention. Therefore, it is possible that the effect sizes were simply too small to be detected in the between-group comparison. Increasing the intensity and duration of the intervention may produce more observable increases in mental quality of life over time compared with the control group. Nevertheless, the null result of the booster session suggests that even a single core session program would be sufficient for enhancing subjective happiness.

Prior research has consistently demonstrated positive responses to positive psychology interventions over a wide range of specific and nonspecific well-being outcomes [51], but their effect sizes could be overestimated, partly because of favorable responses from participants to avoid the embarrassment of having no improvement or to show appreciation for the service providers’ efforts. Our divergent results on subjective happiness and health-related quality of life suggest that social desirability bias was unlikely in the present study. If social desirability bias were present, participants would have responded positively to both measures. The null result of health-related quality of life provides important, although unintended, insight for future studies to include conceptually distinct outcome measures so that the discriminant validity of the intervention can be tested.

### Limitations

This study had several limitations. First, the SF-12v2 health survey, used to assess the effectiveness of the intervention, may be too lengthy and difficult to administer for community-based intervention studies. Future intervention research should consider simple and brief tools that can increase cooperation and reduce the burden, and hence improve the acceptability and validity, especially for participants with busy lives or those from low socioeconomic backgrounds [32]. Second, physical quality of life was assessed using a self-report scale. Although this assessment approach can be administered in large samples and reduces implementation time and cost, the results might be influenced by response and recall bias. Objective or direct methods may provide a more accurate approach to assessing physical health [52]. Third, the small effect size of the intervention may have been due to the brief and preventive nature of the intervention. This type of intervention offered to universal samples has frequently yielded smaller effect sizes than have secondary or tertiary interventions in which there have been typically more room for improvement on the outcomes [53]. The public health approach suggests that even small effects spread over large portions of the population are valuable [54]. Fourth, the findings might be influenced by the incomplete assessment of the total sample that consented to take part in the community programs, because of failure to meet the inclusion criteria or refusal to provide data for research purposes. Although we should have considered rejecting their participation in the community programs, it was not ethical to do so in the contexts of social service units and schools. Fifth, the reduction in sample size due to the exclusion of younger participants aged 6–11 years could have undermined the statistical power. Nevertheless, numerous intervention studies have used smaller samples than that in the present study. Finally, sample size calculation was conducted at an individual-participant level because the data on variance within and between clusters for the calculation of intra-cluster correlation were not available during the planning stage of the study. This could have reduced the statistical power of the study.

## Conclusions

Applying a positive psychology framework to a community-based family intervention is rare in the literature. With the involvement of academic researchers and community practitioners, the findings of this large-scale cRCT provided encouraging, although nondefinitive, evidence to support a brief intervention for improving subjective well-being in the community. The use of cooking and dining as a platform for family activities is culturally relevant for Chinese as well as other populations that emphasize shared cooking and dining practices. Therefore, the findings of this study can be generalized to other collectivistic communities. Modifications to the intervention by targeting not only subjective well-being but also health-related quality of life are warranted.

## Abbreviations

CONSORT, Consolidated Standards of Reporting Trials; cRCT, cluster randomized controlled trial; H1, hypothesis 1; H2, hypothesis 2; HFK I, Happy Family Kitchen; HFK II, Happy Family Kitchen II; ICC, intraclass correlation coefficient; LL, lower limit; MCAR, missing completely at random; SF-12v2, Short Form Health Survey; UL, upper limit

## Additional file

Additional file 1: CONSORT extension for cluster trials checklist. (DOCX 31 kb)
